# Peripheral immune cells and perinatal brain injury: a double-edged sword?

**DOI:** 10.1038/s41390-021-01818-7

**Published:** 2021-11-08

**Authors:** Josephine Herz, Ivo Bendix, Ursula Felderhoff-Müser

**Affiliations:** 1 Department of Paediatrics I, Neonatology and Experimental Perinatal Neurosciences, University Hospital Essen, University Duisburg-Essen, Essen, Germany; 2 Centre for Translational Neuro- and Behavioural Sciences, C-TNBS, Faculty of Medicine, University Duisburg-Essen, Essen, Germany

## Abstract

**Abstract:**

Perinatal brain injury is the leading cause of neurological mortality and morbidity in childhood ranging from motor and cognitive impairment to behavioural and neuropsychiatric disorders. Various noxious stimuli, including perinatal inflammation, chronic and acute hypoxia, hyperoxia, stress and drug exposure contribute to the pathogenesis. Among a variety of pathological phenomena, the unique developing immune system plays an important role in the understanding of mechanisms of injury to the immature brain. Neuroinflammation following a perinatal insult largely contributes to evolution of damage to resident brain cells, but may also be beneficial for repair activities. The present review will focus on the role of peripheral immune cells and discuss processes involved in neuroinflammation under two frequent perinatal conditions, systemic infection/inflammation associated with encephalopathy of prematurity (EoP) and hypoxia/ischaemia in the context of neonatal encephalopathy (NE) and stroke at term. Different immune cell subsets in perinatal brain injury including their infiltration routes will be reviewed and critical aspects such as sex differences and maturational stage will be discussed. Interactions with existing regenerative therapies such as stem cells and also potentials to develop novel immunomodulatory targets are considered.

**Impact:**

- Comprehensive summary of current knowledge on the role of different immune cell subsets in perinatal brain injury including discussion of critical aspects to be considered for development of immunomodulatory therapies.

## Introduction

Perinatal insults resulting in brain injury may affect preterm and also term-born babies. Various noxious stimuli, including perinatal infection yielding excess inflammation, foetal hypoxia, intrapartum oxygen deprivation, hyperoxia, stress from maternal separation and exposure to medication or anaesthesia, increase the risk for long-lasting neurological impairment. In the present review, we will focus on two frequent perinatal conditions, systemic infection/inflammation associated with encephalopathy of prematurity (EoP) and hypoxia/ischaemia in the context of neonatal encephalopathy (NE) and stroke at term. A large body of clinical research investigated biomarkers of injury in order to determine infants at risk for injury and adverse brain maturation. Experimental research concentrated on underlying mechanisms in order to identify targets for neuroprotection and/or induction of repair with the overall aim of personalized therapy for each child.

NE following asphyxia in term infants affects approximately 1.5–3 in 1000 live births in high-income countries^1,2^ and approximately 10–20 of 1000 in low- and middle-income countries^2,3^. Though introduction of hypothermia treatment (HT) into standard clinical practise significantly improved neurodevelopmental outcome, mortality remains high and a significant proportion of infants with NE still suffers from long-lasting motor-cognitive impairment. Children with neonatal acute ischaemic stroke, with an incidence of 7.1–16 in 100,000 live births, are also at high risk for poor neurological outcome^4–7^. The most commonly used experimental model for neonatal asphyxia has been established in rodents by Rice and Vannucci in 1981 with unilateral *common carotid artery* occlusion followed by systemic hypoxia (hypoxia–ischaemia, HI)^8^. The most commonly used neonatal stoke model is the occlusion of the *middle cerebral artery*^9,10^. While initially developed in postnatal day (PND) 7 rodents^8,9^, corresponding to brain development of late preterms, models have been adapted to 9–10-day-old animals to mimic hypoxic and ischaemic insults at term^11–13^. Pathophysiological processes involved in hypoxic/ischaemic brain injury include excitotoxicity, apoptosis, inflammation and subacute white and grey matter injury, resulting in long-lasting brain atrophy mainly affecting cortex, hippocampus and striatum^8,10,13–15^.

In preterm infants suffering EoP, early- and late-onset infections with specific patterns of inflammatory markers have been linked to adverse outcome, including development of motor and/or cognitive impairment, with cerebral palsy as the worst outcome^16–18^. Several experimental models have been developed to mimic inflammation-related development of EoP, e.g., low-dose lipopolysaccharide (LPS) administration on PND3 or repetitive injections of IL-1beta over the first five PNDs. Both injury paradigms lead to a significant impairment of recognition memory, associated with long-lasting alterations of the white matter microstructure^19,20^. Furthermore, previous clinical studies reported higher infection rates in asphyxiated newborns^21^ and an increased risk of brain injury in infected infants with NE^22^. This lead to the development of rodent and large animal models, combining both, systemic inflammation and hypoxic–ischaemic brain injury^23–30^. While the majority of studies applied LPS, the cell wall component of the Gram-negative bacterium *Escherichia coli*, which binds to the Toll-like receptor 4 (TLR4), novel combination models mimicking infections with Gram-positive bacteria, have been recently described. In this regard, the synthetic lipoprotein Pam3CSK4 or infection with live *Staphylococcus epidermidis*, both activating TLR2 signalling are used^24,31–33^. Even though both, TLR4 and TLR2 activation sensitize the brain to increased injury following HI^23,28,29,31–33^, different inflammatory responses are triggered in the brain^34^. Furthermore, responses to living infectious organisms vs. Toll-like receptor ligands may differ. For instance, *Staphylococcus epidermidis* bacteremia induces TLR2-dependent but also -independent pathways, which is supported by the fact that systemic bacterial infection impaired brain development in the absence of bacterial entry into the cerebrospinal fluid (CSF)^35^.

During the perinatal period, both systemic inflammation/infection and hypoxic–ischaemic events induce complex responses in brain-resident immune cells, i.e. microglia, which has been excellently reviewed in several articles^36–39^ and was therefore out of the scope of this paper. Here, we particularly focus on the role of peripheral immune cells in the disease paradigms described above. For a long time, the neonatal immune system has been believed to be immature and impaired in function, with an increased risk for sepsis, reduced responses to certain vaccinations and an increased bias towards differentiation into anti-inflammatory Th2 CD4 T cells^40,41^. However, this concept is challenged by clinical observations of hyper-inflammatory courses of neonatal sepsis^42^. Recent work suggested that neonatal immune responses are not impaired, but rather determined by a tightly regulated physiological immune programming preventing harmful hyper-inflammation in combination with sufficient immunological protection^43^. The potency of the neonatal immune system to elicit a strong reaction to dangerous stimuli is also demonstrated by the fact that neonatal peripheral immune cells participate in sterile tissue inflammation induced by perinatal brain injury^44^, indicating a complex interplay between the peripheral immune and the central nervous system. While the significance of different immune cell subtypes in the development of secondary neurodegeneration has been well established in adult brain injury, e.g. in the context of ischaemic stroke^45^, this research field is still developing in neonatology.

In the present review, we will (1) summarize current knowledge on the role of different leucocyte subsets in perinatal brain injury, (2) provide an overview on different infiltration routes to the injured brain, (3) emphasize the impact of sex, (4) highlight the important aspect of maturational stage and (5) discuss implications for immunomodulatory therapies.

## Divergent role of the various leucocyte subsets in perinatal brain injury

**Fig. 1:**
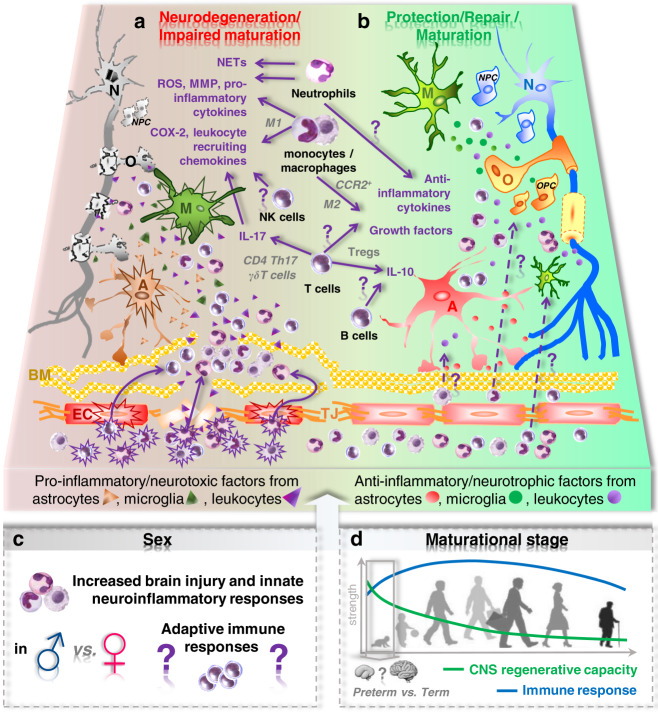
Proposed hypotheses for the divergent role of peripheral leucocytes in perinatal brain injury and influencing factors. **a** Perinatal brain injury induced either by systemic infection and/or by hypoxic/ischaemic events triggers a variety of pathophysiological processes, i.e. endothelial (EC, red/orange), microglia (M, green) and astrocyte (A, brown/red) activation leading to neuronal (N) and oligodendrocyte (O) and progenitor cell (NPC/OPC: neuronal/oligodendrocyte progenitor cell) degeneration and impaired maturation of these precursor cells. Injury-induced activation of EC but also peripheral immune cell (purple) activation (stars) facilitate leucocyte infiltration into the injured brain (swung arrows) via concerted bidirectional molecular interactions involving selectins, integrins and chemokines. However, interactions between leucocytes and ECs also contribute to vascular inflammation and damage to the blood brain barrier composed of ECs connected by tight junction (TJ) proteins, normally tight packed basal membranes (BM) and astrocytic endfeet. Besides contributing to endothelial damage, peripheral leucocytes are supposed to act from the perivascular cuffs by the release of proteolytic enzymes accelerating access of peripheral immune cells to the injured CNS parenchyma, which is further supported by release of chemotactic molecules of activated astrocytes and microglia. Within the CNS, an intense interaction between infiltrated leucocytes and CNS-resident cells leads to the release of a variety of pro-inflammatory and neurotoxic molecules by all involved cell types (triangles). The exact cell source, the time course of expression for each cell type and the relevance for the evolution of brain injury warrants further investigation. So far, only few and very ubiquitous detrimental mechanisms have been proposed as effectors of peripheral immune cells, i.e. neutrophil extracellular trap (NET) formation, reactive oxygen species (ROS) production, increased activity of inflammatory and basal membrane degrading enzymes (e.g. COX-2 and MMPs), and release of pro-inflammatory cytokines. These detrimental effects were specifically ascribed to neutrophils, pro-inflammatory M1 monocytes/macrophages, natural killer (NK) cells and subsets of T cells (i.e. γδ T cells and CD4 Th17 cells). **b** Emerging evidence supports a divergent role of different leucocyte subsets not only promoting damage but also contributing to resolution of inflammation/injury and mediating protection and/or promoting repair. Proposed mechanisms include the release of anti-inflammatory cytokines and growth factors by, for example, regulatory T and B cells as well as protective myeloid cells (e.g. M2 polarized macrophages and CCR2^+^ monocytes). However, most of these hypotheses are based on data derived from adult brain injury models. The contribution of peripheral immune cells to protection and repair in a time-dependent manner following the initial insult is still unclear (question marks). Furthermore, it is important to note that peripheral immune cell subsets have not only been supposed to contribute to repair and regenerative processes but also to be essential for endogenous neurodevelopment, i.e., to support oligodendrogenesis and microglia development. Whether these mechanisms take place from the periphery or the perivascular areas (e.g. meninges and/or CP) and how these signals are mediated is still unclear (dashed arrows with question mark). **c** The general concept about neuroinflammatory/degenerative but possibly also reparative processes mediated by peripheral leucocytes is further challenged by well described sex differences in neurodevelopmental outcome and inflammatory processes. The often-reported increased risk and worse outcome for males is associated with increased innate immune responses in males, supported by the fact that only male mice are protected after depletion of peripheral myeloid cells. Whether such sex dichotomy also applies to cells of the adaptive immune system is still unclear. **d** Another major aspect to be considered according to the hypothesized processes in (**a**) is the maturational stage, since many of them (full arrows with question marks) are still derived from preclinical research in adult brain injury models. However, the immune system and the brain reveal different responses over the life span, reflected by a rather immunoregulatory response in the neonatal stage to limit excess inflammation and an increased capacity of CNS regeneration despite of increased vulnerability of the developing brain. Therefore, concepts cannot be translated unequivocally from the adult organism to the neonatal system. Further challenges, which need to be taken into account for the predicted mechanisms shown in (**a**), are different immune and brain developmental stages in preterm compared to term newborns.

The essential contribution of the peripheral immune system to the development of perinatal brain injury has been initially described in rodent experiments. Splenectomy prior to HI-induced brain injury was associated with pronounced neuroprotection^46^. These data suggested a detrimental role of peripheral immune cells per se. However, emerging evidence supports that perinatal brain injury, induced by HI and/or stimulation of the peripheral immune system, leads to activation and cerebral infiltration of different immune cell subpopulations, mediating either detrimental effects or endogenous neuroprotection depending on the cell type (Fig. 1a, b)^23,32,34,47–55^.

### Neutrophils

The role of neutrophils in perinatal brain injury is poorly understood. Previous reports suggested rare extravasation of neonatal neutrophils into the injured brain, leading to the assumption that neutrophils are clinically less relevant^47,56–58^. However, recent work, including our own, revealed a significant accumulation of neutrophils in the parenchyma of HI-injured brain tissues^50,52^, which was preceded by an acute increase in circulating neutrophils^50^. In support of this, peripheral neutrophilia was observed in the first hours of life after NE in term infants, which was associated with poor neurodevelopmental outcome^59^. Furthermore, clinical evaluations with regard to neutrophil function and phenotype recently demonstrated that neutrophils of infants with NE display a dysregulated inflammatory response, revealed by a hyporesponsiveness to an additional ex vivo inflammatory stimulus, compared to healthy infants^60^, indicating a pre-activated state of neutrophils in NE infants. This is supported by recent analyses of serum and CSF biomarkers associated with activated neutrophils (e.g. interleukin-8 (IL-8)), which were upregulated in moderate or severe NE^61^. While these studies are of utmost importance for prediction of outcome, neutrophil function and phenotype in the brain can hardly be assessed clinically.

Experimentally it was shown that the early increase in the blood was followed by a rapid decline, when neutrophil numbers peaked in the brain^50^, suggesting migration of neutrophils into the brain. Nevertheless, their function in the brain is still a matter of debate. Previous work in models of HI and inflammation-sensitized HI revealed that neutrophil depletion before, but not after, HI is neuroprotective^55,62^, while a recent study demonstrated that depletion, initiated 12 h after the insult provides acute neuroprotection^50^. Several reasons may account for the discrepancy of study results, i.e. differences in outcome measures, methods to evaluate depletion efficiency, species and strategies of depletion. A further, so far underappreciated, aspect is the emerging concept of neutrophil plasticity and heterogeneity. Brain-infiltrated neutrophils reveal an enhanced activation, demonstrated by an increased reactive oxygen species (ROS) production. Furthermore, the frequency of overactivated/aged neutrophils, recently shown to contribute to vascular inflammation and myocardial infarction^63^, was elevated in brain-infiltrated neutrophils compared to blood-derived neutrophils in experimental HI-induced brain injury^50^. Signals that lead to these pronounced changes in neutrophils after entering the brain remain to be elucidated in future studies.

Different effector mechanisms of neutrophils have been suggested in experimental models for neonatal and adult ischaemic brain injury, e.g., formation of neutrophil extracellular traps and disruption of blood brain barrier (BBB) integrity by proteases that degrade basement membranes (e.g. matrix metalloproteinase 9) leading to accumulation of peripheral leucocytes^55,56,64–67^. Previous reports in adult and neonatal ischaemic stroke models as well as inflammation-sensitized HI revealed a pronounced accumulation of neutrophils in the vasculature and the perivascular space^55,56,64,66^, supporting that neutrophils contribute to vascular injury (Fig. 1a). In a model of neonatal stroke, it was further shown that peripheral neutralization of the neutrophil-specific chemokine CINC-1 leads to neutrophil adhesion, which was associated with adverse effects on BBB integrity^56^. Nevertheless, as stated before, a significant amount of neutrophils is also found in the brain parenchyma after hypoxic/ischaemic injury in adult as well as neonatal rodents, suggesting that neutrophils also act from the brain parenchyma behind the BBB and glia limitants (Fig. 1a)^50,52,68^. For example, neuroprotection after neutrophil depletion in neonatal HI was associated with reduced glial activation^50^, indicating that neutrophils promote glial activation thereby contributing to HI-induced neurodegeneration. However, interactions between neutrophils and glial cells are only poorly understood in adult as well as neonatal ischaemic brain injury^69^.

Neutrophils were also the first invaders in the context of the systemic inflammation in the absence of HI, which however depended on the kind of inflammatory stimulus^34^. In spite of similar marked responses in the periphery, activation of TLR2 but not TLR4 signalling was associated with a profound cerebral infiltration of peripheral immune cells, related to release of different leucocyte recruiting chemoattractants^34^. The functional role of neutrophils and their effector mechanisms in models of systemic inflammation-induced perinatal brain injury, regardless of stimulus, is still unknown.

### Monocytes/macrophages

The second major cell population infiltrating the injured perinatal brain are monocytes and macrophages as shown in models of neonatal HI and systemic inflammation, induced by TLR2 activation^34,44,48,49^. Across all disease paradigms, injury-induced monocyte and macrophage infiltration was largely ascribed to upregulation of several monocyte-related chemoattractants (e.g. MCP-1)^23,34,49^. Nevertheless, while a pronounced infiltration of peripheral myeloid cells has been detected in neonatal HI^48,52^, monocyte infiltration was low and the majority of macrophages in acutely injured regions have been identified as microglia in a model of neonatal stroke^70^. Besides differences in models and species, a major reason for these discrepant study results, might be the difficulty to distinguish blood-derived from resident myeloid cells due to shared cell surface epitopes. A commonly used animal model to differentiate between both cell types is the CX3CR1^GFP/+^CCR2^RFP/+^ double transgenic mouse model, in which blood-derived CCR2^+^ monocytes are supposed to express the red fluorescent protein RFP and CX3CR1^+^ resident microglia the green fluorescent protein GFP^71^. Using these mice it was recently shown that neonatal HI leads to infiltration of CCR2^+^ monocytes accompanied by proliferation of CX3CR1^+^ resident microglia^72^. However, this mouse model has its limitations, since CX3CR1 is also expressed on peripheral immune cells (e.g. NK cells). Furthermore, infiltrating CCR2^+^ monocytes downregulate CCR2 after in situ reprogramming as reported in a model of sterile liver injury^73^. Therefore, an elegant transgenic model was recently developed, enabling fate mapping of peripherally derived CCR2 monocytes for at least 62 days after neonatal stroke, uncovering an underappreciated level of long-term monocyte-to-microglia transition after neonatal stroke^74^. Nevertheless, the full extent and spatial distribution of monocyte-derived microglia during long-term brain development are yet to be determined. Furthermore, it remains unclear, whether there is a subacute wave of invading monocytes after neonatal stroke that may be important for resolution of inflammation (Fig. 1b). This is an important question, considering previous reports in adult ischaemic brain injury, where selective targeting of CCR2 in bone marrow‐derived cells and peripheral monocytes caused delayed clinical deterioration, reduced regenerative angiogenesis and impaired long-term behavioural performance^75,76^. Potentially protective properties of CCR2^+^ monocytes were also identified in neonatal rodents exposed to hypoxia–ischaemia at PND3. HI in male CCR2 knockout but not in wild-type mice led to long-term spatial learning deficits associated with reduced numbers of activated macrophages/microglia in the damaged hippocampus^77^.

In addition to the difficulty of discrimination between peripheral and brain-resident myeloid cells, another level of complexity is added by the proposed concept of monocyte/macrophage heterogeneity. Classically activated M1 cells are supposed to promote neurodegeneration and alternatively M2 cells suggested to have anti-inflammatory and regenerative functions (Fig. 1b)^78^. Though, this myeloid cell dichotomy could be verified in vitro^79^, this concept seems to be too simple for the in vivo situation, as previously shown in experimental models of neonatal HI-induced brain injury^80,81^. Despite of the elegant possibility to perform selective analysis on ex vivo sorted myeloid cells from injured brains in different perinatal brain injury models, including traumatic brain injury, HI and perinatal stroke^28,80,82–84^, effects of peripheral monocytes/macrophages can hardly be distinguished from effects of resident microglia. Even though fluorescent-activated cell sorting (FACS)-based techniques may offer alternative strategies to separate both subsets (different CD45 expression), single-cell transcriptomic analyses may offer novel possibilities to identify specialized myeloid cell subsets. Besides cellular characterization and fate mapping, interventional approaches to selectively analyse the functional relevance of either peripheral or resident myeloid cells remain a major hurdle in neonatal research. While in adult brain injury models, the specific role of brain-resident microglia was mainly assessed by pharmacological inhibition of the colony-stimulating factor 1 receptor (CSF1R)^85–87^, this is not applicable in neonates due to long treatment periods needed. However, an elegant transgenic mouse model was developed, enabling rapid depletion of microglia, which was associated with aggravated HI-induced brain injury^88^. With regard to peripheral monocytes and macrophages, chlodronate liposomes might provide an efficient and selective strategy to deplete peripheral myeloid cells^86^, which needs further investigation in neonatal models.

### Lymphoid cells of the innate immune system

Natural killer (NK) cells and gamma delta (γδ) T cells are increasingly recognized as potential modulators of neonatal brain injury. Clinical evaluations in NE demonstrated higher γδ T cell numbers and frequencies in neonates with NE and children with cerebral palsy compared to age-matched controls^89^. Furthermore, NK and γδ T cells from NE neonates revealed a more rapid inflammatory cytokine response upon ex vivo stimulation, suggesting that they were previously primed or activated. Interestingly, these alterations persisted into school age^89^.

#### NK cells

Experimentally, it was shown that NE caused by HI leads to pronounced infiltrations of NK cells even exceeding those of T cells^46^. Furthermore, increased brain injury after pharmacological T cell depletion was associated with a 3.5-fold increase of NK cells in HI-injured brain tissues^48^. However, data on the functional role of NK cells in neonatal brain injury are limited. HI-induced increases of the pro-inflammatory mediator cyclooxygenase 2 (COX-2) were diminished after interfering with NK cells by knocking down CD161, suggesting a pro-inflammatory role of NK cells in HI-induced brain inflammation and associated injury (Fig. 1a)^46^. While a causal relationship is still missing in neonates, NK cell ablation experiments in an adult brain ischaemia model demonstrated increased brain injury in NK cell-depleted mice, which was independent of T, NKT and B cells^90^. As a potential driver of detrimental effects recent work suggested that astrocytic IL-15 could aggravate post-ischaemic brain damage via propagation of NK cell-mediated immunity^91^.

While depletion of NK cells significantly ameliorated depression-like behaviour in adult LPS-treated mice, which was related to the chemotactic role of NK cells to recruit neutrophils^92^, the role of this subset in systemic inflammation-induced perinatal brain injury is unknown.

#### γδ T cells

Large numbers of innate γδ T cells were detected in post-mortem brain tissues of preterm human infants and in tissues of small and large animal models of perinatal HI^93^. Depletion of γδ T cells provided neuroprotection of HI-induced brain injury in preterm-equivalent rodents^93^, suggesting a detrimental role of this immune cell subset, which is supported by findings from adult injury models^94^. IL-17 released by γδ T cells is supposed to be the major detrimental effector molecule, activating metalloproteinases and inducing expression of pro-inflammatory and neutrophil-attracting cytokines (Fig. 1a)^94^. Therefore, modulating the activity of γδ T cells in perinatal brain injury might represent a novel therapeutic target. Intriguingly, antibiotic treatment-induced interstitial dysbiosis led to a reduction of meningeal IL-17^+^ γδ T cells associated with reduced ischaemic injury in an adult brain ischaemia model, uncovering a previously unrecognized role of these cells in the gut–brain axis^95^. The role of the neonatal microbiome for neurodevelopment is increasingly acknowledged^96^. The causal relationship between remote immune responses in the intestine and the development of perinatal brain injury is not well understood. However, the postnatal period is particularly critical for the development of microbiota composition and immune homoeostasis^97,98^. For instance, a TLR5-dependent regulatory circuit of neonatal bacterial colonization acting solely during the early neonatal period was recently reported to influences life-long microbiota composition^99^. Future research has to clarify the impact of microbiome-associated changes in the peripheral immune system on the development of perinatal brain injury.

With regard to systemic inflammation, it was shown that this innate T cell subset but not adaptive αβ T cells contribute to sepsis-induced white matter injury and subsequent motor function abnormalities in early life^100^.

### Lymphoid cells of the adaptive immune system

Emerging evidence of clinical and preclinical studies indicate an important role of peripheral T and B cells in the development of perinatal brain injury^44,46–48,53,54,101–104^. The suggested dynamics of T and B cell infiltration observed at 1 and 7 days and an additional emergence three months after injury^47,53,101,103^ make them amenable to therapeutic intervention. This is supported by previous work in mature lymphocyte-deficient *Rag1*^−*/−*^ mice that were protected from HI-induced white matter loss in 5-day-old mice^103^. However, a very recent report using severe combined immunodeficient (SCID) mice, which also lack functional T and B lymphocytes, did not observe differences in HI-induced brain injury in 12-day-old animals^105^. This discrepancy might be ascribed to different transgenic mouse strains, but most likely also to different developmental stages. Nevertheless, common to both studies is the lack of both, T and B cells, impeding clear conclusions about the specific contribution of each cell type.

#### T cells

To dissect the specific role of T cells adoptive transfer experiments with T and B cells in lymphocyte-deficient animal models are needed, as recently demonstrated in a model of adult stroke, where transfer of T cells but not B cells exacerbated infarction in Rag^−/−^ mice^106,107^. However, adoptive transfer experiments via the intravenous route are challenging in neonatal animals. An alternative approach is the pharmacological modulation of systemic lymphoid cell subsets, e.g., by Fingolimod (FTY720), which is an immunomodulatory sphingosin-1-phosphate analogue, blocking egress of lymphocytes from lymphoid organs leading to reduced lymphocyte counts in the circulation and thus in the injured brain^108,109^. Own previous experimental work in neonatal rodents revealed that FTY720 particularly reduces circulating T cells but not B cells^48^. Interestingly, FTY720 treatment of HI-injured mice aggravated brain lesions^48^. These results suggest an endogenous protective function of neonatal T cells in HI-induced perinatal brain injury. Indeed, antibody-mediated T cell depletion also enhanced brain injury in our 9-day-old mouse model^48^. Furthermore, FTY720 blocked the selective endogenous infiltration of immunosuppressive/regulatory T cells (Treg)^48^, which are supposed to confer endogenous neuroprotection in adult brain injury (Fig. 1b)^110^. Therefore, the particular lack of neuroprotective Tregs upon FTY720 treatment may account for increased brain injury after depletion of the total T cell population. This is supported by previous work in an ovine preterm global HI model reporting that mesenchymal stem cells (MSCs) for neuroprotection induce peripheral T cell tolerance^111^.

The mode of action of T cells apparently depends on the kind of injurious stimulus, since it was previously shown that in contrast to ischaemic brain injury the general lack of αβ T cells does not influence LPS-induced white matter loss^100^. However, pro-inflammatory αβ T cell subtypes, i.e., CD4 Th17 cells are supposed to play an important role in the combined setting of peripheral inflammation and HI, demonstrated by an increased systemic frequency of pro-inflammatory Th17 cells compared to healthy controls^54^.

#### B cells

Little is known about B cells. Neonatal HI leads to an upregulation of genes involved in B cell proliferation and differentiation^112^. Previous work further revealed that enhanced HI-induced brain injury in adenosine A1 receptor knockout mice was associated with reduced activation and infiltration of IL-10-producing B lymphocytes, indicating a protective function of these cells in this setting^104^. In support of this it was shown that regulatory B cells control inflammation in neonatal mice via production of IL-10 following TLR9 stimulation^113^.

## Infiltration routes of peripheral immune cells

### Blood brain barrier

Different barrier systems have been described to protect the brain against the invasion of harmful pathogens and leucocytes, thereby contributing to the so called “immune privilege of the brain”. Traditionally, these barriers were supposed to be immature during development, contributing to increased vulnerability of the immature brain. However, emerging evidence suggests that these barriers are well developed to combat injurious insults^114^. In models of neonatal stroke, it was reported that blood brain barrier (BBB) integrity is more preserved in neonates compared to adults^56^. However, common to all perinatal insults, discussed in this review, is a general disturbance of BBB integrity compared to the healthy brain, as excellently reviewed by Mallard et al.^114^. Even though the detailed underlying molecular and cellular mechanisms are only partially understood, increased BBB injury is associated with an increased accumulation of peripheral immune cells in the injured brain. Suggested mechanisms involve damage to tight junction proteins^115,116^, upregulation of chemokines^23,117^ and endothelial adhesion molecules^118–120^ and disruption of the normally tight packed basal membranes^50^ (Fig. 1a).

### Blood–CSF barrier

In addition to the traditional infiltration route through the impaired BBB, further anatomical interfaces as potential gateways to the brain are increasingly recognized in adult and neonatal brain injury. These include the choroid plexus (CP) through CSF and the subarachnoid space through meningeal vessels^121–123^. Recently the CP was suggested to be an important infiltration route of monocytes and neutrophils in the context of neonatal stroke, supposed to be mediated through CX3CR1-CCR2-dependent mechanisms^124^. In the context of systemic perinatal inflammation, it was shown that TLR2 activation leads to strong neutrophil and monocyte infiltration to the CSF through the CP associated with TLR2-specific alterations of chemotactic molecule expression and basement membrane remodelling^33,34^. First therapeutic approaches have been tested, i.e. *N*-acetylcysteine blocked neutrophil migration across the endothelium of choroidal stromal vessels and the epithelium, both forming the blood–CSF barrier^117^.

### Alternative routes

Further alternative infiltration routes have been suggested in adult brain injury models. A recently discovered route for neutrophil migration into the ischaemic brain is displayed by direct connections from the skull bone marrow to the brain^125,126^. However, the presence of these structures in the neonatal skull has not been investigated. Other potential gateways may be lymphatic vessels in the meninges^127^, recently suggested to be used for drainage of CSF components and lymphocyte migration to the draining lymph nodes during chronic neuroinflammation^128^. Whether and how these alternative infiltration routes and immunological communication hubs orchestrate the inflammatory response in the injured neonatal brain is still unknown. Further research in this direction may uncover new possibilities for selective intervention.

## Impact of sex

Male sex is a well-established epidemiological risk factor for poor neurodevelopmental outcome and increased innate neuroinflammatory responses after perinatal brain injury (Fig. 1c)^129–134^. The risk for perinatal stroke or to experience an HI insult is supposed to be higher in males combined with more severe neurological deficits^133,135,136^. Furthermore, clinical and experimental studies suggested a sexual dimorphism in response to neuroprotective therapies, e.g. hypothermia^130,133,137^. Infections, both during pregnancy and in childhood also contribute to the sex-dependent risk for the development of long-term neurological sequelae, specifically for neuropsychiatric disorders^138^.

Preclinical studies demonstrated an increased HI-induced brain atrophy in males^139^, associated with differences in long-lasting immune responses, revealed by elevated circulating levels of tumour necrosis factor (TNF)-α in adolescent male mice and a more pronounced lymphocyte infiltration in injured brain hemispheres accompanied by reduced neurogenesis^129^. Interestingly, sex differences become particularly evident in the phase of secondary neurodegeneration and inflammation. This is supported by Mriza et al.^132^, who observed more severe damage and increased pro-inflammatory cytokine expression in brain tissues of males 3 days but not 1 day after HI. Similarly, males exhibited larger tissue loss than females 3 months after experimental neonatal stroke, but infarct volumes did not differ 3 days after ischaemia^84,140^. With regard to systemic inflammation, a similar sex dichotomy was described, i.e., infection with *Staphylococcus epidermis*, activating TLR2, potentiates HI-induced brain injury in male but not in female animals^31^. There are, however, first indications, that activation of TLR3 with the viral mimetic Poly IC increases early extrinsic apoptotic pathways in neonatal female but not in male rodents, though males revealed increased inflammatory responses (i.e. astrogliosis and IL-6 expression) in the brain^141^.

Sex differences in cell death pathways and inflammatory responses have been discussed^130^, but their origins in perinatal injury are yet poorly understood. While a large body of evidence points to an important role of microglia^84,138,142,143^, the impact of sex on peripheral leucocytes is not well explored. This is, however, important, considering pronounced physiological differences between neonatal male and female immune responses, reflected by more robust innate immune responses in males, while females have higher CD4/CD8 T cell ratios^144^. A major driver of the well-known sex differences in immunity are sex hormones with a plethora of effects on immune system function^144^. For example, oestrogens promote the expansion of Tregs and induce Th2 responses^145^, accompanied by a decreased production of IL-17 by pro-inflammatory Th17 cells^146^. Even though, hormonal influences on neonatal immune cells and consequently on outcome after perinatal brain injury cannot be entirely excluded, lacking sex-hormone differences before puberty indicate intrinsic/genetic differences^132,144^. Preclinical reports demonstrating no sex differences in hormone levels but differences in inflammatory responses and outcome^129,132^ support that intrinsic mechanisms may account for divergent susceptibility to the development of neurological deficits. For example, first preclinical studies revealed an increased myeloid cell infiltration in males following neonatal stroke^84^. Furthermore, antibody-mediated depletion of peripheral myeloid cells provided neuroprotection only in male mice^52^. The impact of sex on cells of the adaptive immune system in perinatal brain injury is largely unknown. Mirza et al.^132^ showed an increased infiltration of lymphocytes in HI-injured brains of males. However, cells types were not discriminated. Considering the complex and divergent functions of different lymphocyte subtypes (see sections “Lymphoid cells of the innate immune system” and “Lymphoid cells of the adaptive immune system”, Fig. 1a, b), further in-depth analyses of specific cell types are needed. Potentially important cells might be regulatory T cells (Treg), for which sex-dependent differences in functionality and phenotype have been reported in different adult disease paradigms^144^. Besides sex-hormone-dependent mechanisms, differences in the transcriptomic and metabolic profile between female and male Tregs have been recently discussed in the context of chronic intestine and visceral adipose tissue inflammation^147,148^. There are no such studies in adult stroke models available since only male mice were used in the majority of studies. In the context of perinatal brain injury, studies on sex-specific Treg responses are also pending.

In view of the huge amount of epidemiological evidence over more than two decades, preclinical in vivo research is now beginning to include sex-stratified analyses across a huge variety of perinatal injury models. However, our understanding of potential functional and phenotypical sex differences in cells of the peripheral immune system and their impact on the development of perinatal brain injury is still in its infancy.

## Impact of maturational state

The uniqueness of the neonatal immune system is demonstrated by significant differences in quantity and function of innate and adaptive immune cells compared to adults (Fig. 1d), as comprehensively summarized by Tsafaras et al.^41^. While the impact of these pronounced differences is increasingly acknowledged in immunological research, including sepsis and vaccination research, their impact on neurodevelopmental disorders is still in its infancy. This is, however, of utmost importance, since the brain is still developing, which makes it more vulnerable to noxious stimuli on the one hand. On the other hand, the maturing brain is supposed to have an increased regenerative capacity, offering unappreciated opportunities for intervention, which can hardly be derived from research in adult brain injury (Fig. 1d). Another level of complexity is added by marked differences in immune responses and brain injury mechanisms between injury affecting preterm and term-born babies (Fig. 1d). So far, systematic comparative analyses for each of the discussed leucocyte subsets with regard to different immune cell functions comparing either perinatal vs. adult brain injury or preterm vs. term-born are rare. Therefore, we will focus on two main subsets of immune cells in this chapter, i.e., neutrophils and T cells.

### Neutrophils

Even though neonatal neutrophils are believed to be immature and impaired in function compared to adult neutrophils (e.g. weaker responses to infection, less migratory capacity, less ROS production)^149–151^, they infiltrate the injured brain early after neonatal HI^50,52^. Similar detrimental effector mechanisms appear to be involved in adult and neonatal ischaemic brain injury, contributing to injury evolvement in the early disease phase, e.g., through ROS production^50^ and interaction with the vasculature^14,55,56^ (Fig. 1a). Nevertheless, the impact of neutrophils also seems to depend on the maturational stage in the neonatal phase. For instance, in rodents with inflammation-sensitized HI-induced brain injury, an insult at PND1 (corresponding to preterm birth) did not lead to accumulation of neutrophils, while pronounced neutrophil infiltration was observed in the same experimental setting, when induced at PND12 (corresponding to term birth)^23^. These age-dependent differences correlated with significant and longer-lasting cerebral expression of the neutrophil-specific chemokine CINC-1 in term-born equivalent rodents^23^.

In addition to timing of the insult, neutrophils may have a divergent role in different time frames after the initial insult. Until now, the majority of adult and neonatal studies focused on the role of neutrophils in the acute injury phase, neglecting effects on endogenous repair mechanisms occurring days to weeks later^50,52,55,62,107,152^. Recent experimental findings showed a second infiltration of total myeloid cells 7 days after HI^52,53^, when regeneration and repair are initiated^153^. Considering that perinatal brain injury induced inflammatory processes last into the secondary and tertiary disease phase^154^ overlapping with endogenously induced regenerative processes, myeloid cells might contribute to endogenous repair mechanisms (Fig. 1b), e.g., be the release of growth factors and proangiogenic factors, as recently described in adult brain injury models^155–159^. In support of this, it was recently shown that neutrophils promote regeneration in an adult model of optic nerve regeneration and spinal cord injury^159^.

### T cells

In contrast to adult ischaemic brain injury^160^, peripheral lymphocyte and specifically T cell depletion by FTY720 treatment in HI-injured mice aggravated brain lesions^48^. These results do not only confirm previous reports about questionable translation from adults to neonates^10^, but also suggest an endogenous protective function of neonatal T cells in HI-induced perinatal brain injury. Compared to adults, neonatal T cells reveal an increased bias towards anti-inflammatory T cell subsets^40^, which might provide an endogenous protective mechanism to limit excess inflammation in the neonatal organism. The selective blockade of endogenous infiltration of Tregs in FTY720-treated animals^48^ may explain differences in outcomes between neonatal and adult mice. This is supported by the fact that neonatal Tregs are supposed to have an increased activation state and immunosuppressive function compared to adults^161–163^. Furthermore, T cell function also seems to depend on the maturational stage already in the neonatal phase. Studies indicating a detrimental role of T cells preferentially investigated preterm-equivalent HI models using PND4 and PND7 rodents^100,103^ or transient umbilical cord occlusion^111^. In contrast, analyses in term-born equivalent PND9 and PND12 mice, either lacking both, T and B cells, or with selective T cell depletion showed no differences or indicated endogenous neuroprotection by T cells, respectively^48,105^. Taking into account that the stage of white matter development and axonal outgrowth in the rodent CNS between PND1 and PND7 corresponds to 23–36 weeks gestation in human brain development^164^, infiltrated T cells may impede vulnerable CNS maturation processes in this critical period^165^. In contrast, in the term-equivalent brain of PND9-PND12 rodents, T cells may rather protect from HI-induced damage of mature neural cells. Physiological changes in the peripheral immune system between PND5 and PND9 rodents revealed by a continuous increase of lymphocytes and a decrease of neutrophils within the first 2 weeks of life may also play a role^44,166^. In spite of lacking systematic ontogenetic characterization of T cell subpopulations, first clinical evaluations demonstrated that extreme prematurity is associated with increased Treg frequencies and suppressive activity compared to Tregs of term neonates^167,168^.

Similar to neutrophils, T cells and their subsets may obtain different functions in different time frames after the initial insult. Experimental studies showed that there is a second wave of T cell infiltration 7 days after HI^53,101^, which might be important for endogenous repair and neurogenesis on the one hand. On the other hand, the second accumulation of CD4 T cells 7 days after HI was also associated with enhanced expression of RORγt, a transcription factor specific for the pro-inflammatory CD4 Th17 T cell subset^101^. The contribution of different T cell subpopulations to tertiary mechanisms of perinatal brain injury is still unknown and warrants further investigation.

Taken together, further clinical and experimental studies are needed to dissect the role of the different leucocyte subsets in different time frames after injury but also for different timings of the insult (i.e. preterm vs. term). This would allow definition of selective therapeutic windows for modulation of specific leucocyte subsets to enable protection, repair, and restoration of physiological brain development.

## Implications for treatment

First clinical evaluations and experimental intervention studies identified the peripheral immune system as possible target for novel therapeutic strategies. The detrimental role of early neutrophils and myeloid cells was uncovered by selective depletion of these cell types^50,52^, which however bears severe risks for neonates and is therefore no therapeutic option. According to previous studies in adult stroke models and perinatal brain injury caused by intrauterine chorioamnionitis, an alternative strategy might be the inhibition of peripheral leucocyte infiltration, e.g., by blockade of adhesion molecules and chemokine receptors^48,107,169^. However, a better understanding of the increasingly recognized plasticity and heterogeneity of myeloid cell types may provide new opportunities to modulate immune cells in the periphery towards protective cell types, as previously suggested in models of optic nerve crush, spinal cord injury and stroke^155,159^.

### Hypothermia and immunomodulation

Neuroprotection induced by delayed neutrophil depletion 12 h after injury suppose larger therapeutic windows of immunomodulatory therapies compared to standard therapies like hypothermia (HT). Therefore, interfering with peripheral immune responses may overcome current limitations of HT. However, potential interaction effects between anti-inflammatory HT and immunomodulatory therapies should be considered. In the clinical setting HT induces a transient leukopenia between 60 and 72 h and persisting low white blood cell counts after rewarming were associated with poor outcome^170^. Preclinical data systematically investigating the impact of HT on the fate and function of different leucocyte subsets is still missing. Even though recent work with SCID mice lacking T and B cells did not indicate any adverse interaction effects^105^, further work is needed to evaluate either interaction or potential synergistic effects between HT and immunomodulatory therapies.

### Impact of disease aetiology and time point of intervention

Post-hypoxic neutrophil depletion provided acute neuroprotection in our work in a mouse model of pure HI^50^, while being less efficient in LPS-sensitized HI^55^. Furthermore, FTY720 had detrimental effects in pure HI^48^, while being protective in inflammation-sensitized HI^54^. Discrepant study results may be related to different injury severities, i.e. animals in the combined setting revealed up to 40% tissue loss in the cortex while only 10% cortical tissue loss were observed in pure HI^48,54^. Injury severity seems particularly important for the extent of neuroinflammatory responses but also for the efficiency of immunomodulatory therapies, as already reported in adult models of ischaemic stroke at the example of FTY720 treatment^160,171–173^. Besides different injury severities, disease aetiology might play a major role in this context, since systemic activation of the immune system by LPS strongly alters the peripheral immune system, as previously shown^34^. Therefore, immune cell function in the HI-injured brain most likely differs compared to sterile inflammation induced by the sole HI event. However, a systematic comparison of leucocyte subset function in the brain between both entities is still lacking. In addition to the experimental setting and disease aetiology, the time point of intervention might affect treatment outcome. To date, the majority of studies focused on the acute role of peripheral immune cells, e.g., by initiation of cell depletion prior to or shortly after HI. While these approaches target the initial influx of cells, the relevance of a second infiltration peak 7 days after HI observed for myeloid cells and T cells^52,101^ can hardly be predicted by these experimental approaches. Furthermore, transgenic mouse models (Rag^−/−^, SCID, αβ T cell receptor^−/−^, γδ T cell receptor^−/−^) have a permanent depletion of specific T cell subsets or of both, T and B cells^93,100,103,105^. In the majority of these studies, analyses were performed 7 days after the insult. Therefore, modulation of the early, the late or of both infiltration time points might have led to the observed study results. The same holds true for a single FTY720 application shortly after HI, which led to a sustained depletion for at least for 7 days^48^. Therefore, further studies, selectively targeting distinct time points after the insult will be important, considering the different time windows of opportunity.

### Immunomodulatory effects of stem-cell based regenerative therapies

In addition to selectively targeting cells of the peripheral immune system, multimodal therapies, indirectly modulating peripheral leucocytes, i.e., by MSC and MSC-derived extracellular vesicles (EV) represent promising therapeutic approaches. In a variety of classically immunological but also other disease paradigms, MSCs have been described to modulate innate and adaptive immune responses, thereby reducing tissue injury and creating an environment supporting tissue repair and regeneration^174,175^. With regard to perinatal brain injury, a tremendous amount of reports revealed the neuroprotective potential of MSCs and MSC-EVs^19,81,111,119,176–183^. However, the underlying mode of action of these stem-cell-based regenerative therapies are poorly understood. While the majority of studies particularly focused on cell damaging and regenerative effects in the brain, MSCs’ and MSC-EVs’ immunomodulatory effects in the periphery have been rarely addressed in perinatal brain injury models. Intravenous MSC administration in an ovine global HI model revealed that protective effects were associated with a diminished HI-induced splenic involution, increased T cell tolerance and reduced cerebral T cell infiltration^111,177^. These studies suggest that modulation of peripheral immune cells may contribute to stem-cell-based protective and regenerative effects. With regard to clinical translation, administration routes have been adapted to intranasal (i.n.) applications of MSCs and MSC-EVs, particularly in rodent models^119,176,182^. While this administration route provides similar protective effects, the mode and place of action may differ. Even though it is supposed that i.n. application of MSCs and MSC-EVs reach the brain easier and may rather directly modulate injurious and regenerative processes in the brain^176,184–186^, the potential impact on the periphery via this route is not well explored. However, first analyses of serum samples from rodents treated with MSCs via the intranasal route indicate that i.n. administration of stem cells may also modulate peripheral immune responses^119^. A major challenge in this research field will be to dissect immediate targets in the brain and indirect mechanisms in the periphery to improve efficiency of stem-cell-based therapeutic approaches.

## Conclusion and perspectives

The pathogenesis of perinatal brain injury is complex and multifactorial. In this review, we focused on brain injury induced by systemic inflammation and hypoxic/ischaemic insults in the perinatal period. Though different in aetiology, all of them are associated with neuroinflammatory processes involving the peripheral immune system. Against the common concept of an immature and impaired immune system, an emerging body of evidence suggests a tightly regulated responsiveness of neonatal leucocyte subtypes to injurious stimuli associated with perinatal brain injury. Modulation of peripheral immune responses may offer novel opportunities for intervention due to easier access compared to the target organ, the brain. However, our knowledge on the contribution of different leucocyte subsets to acute neurodegeneration but also repair is too immature to propose novel therapeutic strategies. This will require further systematic analysis in the periphery and in the injured brain, experimentally and clinically. Experimentally, the majority of findings is based on studies in rodents, which need to be verified in large translational animal models and clinical studies. While clinical correlation analyses between peripheral immune responses and neurological outcome will provide utmost important predictive values, experimental studies may uncover cause-and-consequence interrelations.

A major challenge is to improve our understanding about the plasticity and heterogeneity of different leucocyte subsets depending on several factors, like sex, time window of assessment, tissue environment and aetiology of disease. Furthermore, time and place of immune cell priming needs to be elucidated. In addition to the discussed gut–brain axis, interactions between lung and brain may also shape leucocyte phenotype and function since the plethora of noxious stimuli associated with perinatal brain injury and long-term neurological deficits do not exclusively affect the brain. Therefore, a major need will be the identification of direct cellular targets of different leucocyte subtypes to answer the question, whether they act already in the periphery modulating other peripheral immune cells or whether they mediate their effects directly in the brain, e.g., through interaction with other CNS cells like microglia^187^. Moreover, in view of previous work, aiming to identify inflammatory biomarkers for prediction of outcome it needs to be elucidated to which extent peripheral immune activation is cause or consequence of perinatal brain injury. While increased levels of IL-6, C-reactive protein and myeloperoxidase in umbilical cord serum were associated with preterm birth, these did not correlate with poor neurodevelopmental outcomes^188^. However, increased IL-6 levels before initiation of HT in term-born infants with HIE were associated with adverse short-term MRI outcome^189^. With regard to peripheral cellular responses, it was suggested that, on the one hand, high neutrophil numbers after HIE are associated with poor outcome^59^. On the other hand, HT-induced reductions of white blood cell counts were also associated with worse outcome^170^. According to this, reversal of stroke-induced lymphopenia in a model of adult ischaemic brain injury was associated with enhanced neuroregeneration and functional improvement^190^. These data demonstrate the complexity of the topic and the urgent need for an in-depth understanding of the delicate balance between protective and detrimental peripheral immune responses. When interfering with the immune system, it should be taken into account that peripheral immune cells may not only promote tissue injury but also contribute to regeneration and physiological brain development (Fig. 1b), as elegantly shown at the example of CD4 T and B cell subsets^191,192^. Another aim of future work to answer the difficult question of cause and consequence will be to clearly distinguish the role of peripheral immune cells in (infectious) systemic inflammation-induced brain injury and sterile tissue inflammation, caused by ischaemic events. First preclinical data indicate that both insults may activate different inflammatory pathways. For example, systemic endotoxin exposure robustly induced TLR2 expression in the brain and increased levels of several inflammatory cytokines/chemokines, while TLR2 expression and inflammatory responses were reduced in the paradigm of sterile inflammation induced by intracerebral IL-1β injection or neonatal stroke^49^.

Another important challenge is the understanding of timing and consequences of leucocyte priming during the perinatal period for long-term neurodevelopment. In spite of the overwhelming data linking perinatal inflammation to long-term neurological impairment^37,138,193^, the majority of research focused on early and late cytokine responses and the important role of microglia. The impact of peripheral immune cell priming by noxious stimuli during the perinatal period on the development of long-term neurological disorders is only poorly understood. Recently, a causal link between increased neonatal monocyte infiltration caused by the combination of maternal inflammation and postnatal HI and development of autistic-like behaviours in the offspring was demonstrated^194^. Furthermore, experimental chorioamnionitis, a leading cause of preterm birth, resulted in increased cerebral neutrophil activation at PND7, which could be reversed by inhibition of early neutrophil infiltration leading to amelioration of grey and white matter injury at juvenile age (i.e. PND21)^169^.

Taken together, in spite of the high translational potential, the present review demonstrates that this topic is a novel research field, just beginning to develop. Both, detailed preclinical research, elucidating the causal interrelationship between the peripheral immune and the central nervous system and correlative clinical evaluations will help to improve our understanding of factors contributing to peripheral immune cell plasticity and function in a time-dependent manner. These upcoming findings may offer possibilities to develop novel therapies for treatment of perinatal brain injury and for prevention of long-term neurological disorders.
